# The 3′ Region of the Chicken Hypersensitive Site-4 Insulator Has Properties Similar to Its Core and Is Required for Full Insulator Activity

**DOI:** 10.1371/journal.pone.0006995

**Published:** 2009-09-10

**Authors:** Paritha I. Arumugam, Fabrizia Urbinati, Chinavenmeni S. Velu, Tomoyasu Higashimoto, H. Leighton Grimes, Punam Malik

**Affiliations:** 1 Division of Experimental Hematology and Cancer Biology, Cincinnati Children's Hospital Medical Center, Cincinnati, Ohio, United States of America; 2 Division of Hematology-Oncology, Cincinnati Children's Hospital Medical Center, Cincinnati, Ohio, United States of America; 3 Division of Immunobiology, Cincinnati Children's Hospital Medical Center, Cincinnati, Ohio, United States of America; University of Minnesota, United States of America

## Abstract

Chromatin insulators separate active transcriptional domains and block the spread of heterochromatin in the genome. Studies on the chicken hypersensitive site-4 (cHS4) element, a prototypic insulator, have identified CTCF and USF-1/2 motifs in the proximal 250 bp of cHS4, termed the “core”, which provide enhancer blocking activity and reduce position effects. However, the core alone does not insulate viral vectors effectively. The full-length cHS4 has excellent insulating properties, but its large size severely compromises vector titers. We performed a structure-function analysis of cHS4 flanking lentivirus-vectors and analyzed transgene expression in the clonal progeny of hematopoietic stem cells and epigenetic changes in cHS4 and the transgene promoter. We found that the core only reduced the clonal variegation in expression. Unique insulator activity resided in the distal 400 bp cHS4 sequences, which when combined with the core, restored full insulator activity and open chromatin marks over the transgene promoter and the insulator. These data consolidate the known insulating activity of the canonical 5′ core with a novel 3′ 400 bp element with properties similar to the core. Together, they have excellent insulating properties and viral titers. Our data have important implications in understanding the molecular basis of insulator function and design of gene therapy vectors.

## Introduction

Chromatin insulator elements are boundary elements that separate active transcriptional domains in the genome to allow differential regulation of genes, and prevent the spread of heterochromatin towards active transcriptional units [1], [2]. The importance of insulator elements have recently come to light with observations of silencing of transgenes [3], [4], [5], [6], or inadvertent activation of surrounding cellular genes [7], [8] that have occurred with randomly integrating viral vectors and have led to severe adverse events in the X-linked severe combined immunodeficiency (X-SCID) [9] and chronic granulomatous disease (CGD) [8] gene therapy trials. Vectors have since been modified for safety, with a self-inactivating (SIN) design to delete the viral enhancers and promoters and incorporate endogenous cellular promoters [10], [11], [12], [13], [14].

Chromatin insulator elements can confer an important safety feature to these randomly integrating vectors. A DNase hypersensitive fragment upstream of the chicken β-globin gene locus, the chicken hypersensitive site-4 (cHS4) is a prototypic insulator that has been extensively characterized. It has been shown to have enhancer-blocking activity [14], [15], [16], and prevent proviral silencing to provide uniform expression that is independent of the site of integration and resists transgene silencing (barrier activity) [17], [18]. Uniform expression unaffected by enhancers/repressors in surrounding chromatin allows lower vector copies for a therapeutic effect. Indeed, ‘uninsulated’ lentiviral vectors have variable expression and require multiple copies for a therapeutic effect [19]. We have showed that the cHS4 insulator provides uniform gene expression from lentivirus vectors that is resistant to chromatin position effects. This results in a 2-fold higher overall β-globin expression [17] to correct the human β-thalassemia major phenotype [20], Insulated gamma-retrovirus vectors also resist proviral silencing [18], [21], [22] Moreover, the cHS4 insulator reduces insertional activation of cellular genes [15], [16], [23]. Despite these beneficial effects, the large 1.2 kb cHS4 is not favored in viral vectors, due to its deleterious effect on vector titers [24].

Two distinct and separable insulator activities of cHS4 are confined to the 5′ 250 bp termed the “core” [25], [26]. Specifically, enhancer blocking activity has been mapped to a 90-bp fragment containing a CTCF (CCCTC-binding factor)-binding motif [27] and barrier activity mapped to USF-1/2 motif in the core [28], [29], [30]. CTCF sites are distributed genome-wide in intragenic regions, and conserved across species [31], [32], [33], [34], [35]. Despite these well characterized motifs, the cHS4 core does not show significant insulator activity in viral vectors. We therefore performed a structure-function analysis of cHS4 and studied the epigenetic changes that accompany “insulation” of transgenes. We found unique insulator properties in the distal 3′ 400 bp of the cHS4 insulator, furthest from the canonical core, which when combined with the 5′ core, restored full insulator activity and yet retained good viral titers.

## Results

### Vector constructs and experimental design

**Figure 1:**
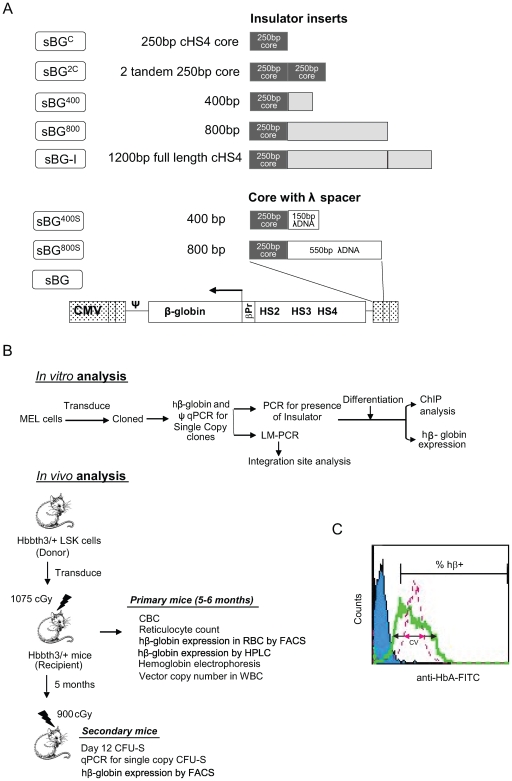
Vector constructs and experimental design. A. Self-inactivating (SIN) lentiviral vector carrying the hβ-globin gene and the HS2, HS3 and HS4 of the locus control region is shown as sBG. Using this backbone, a series of vectors were generated to incorporate either the cHS4 5′ 250 bp core, 2 tandem repeats of the core, 5′ 400 bp or 5′ 800 bp of cHS4, and the full-length 1.2 Kb cHS4 insulator. Vectors sBG^400S^and sBG^800S^ carry in addition to the core inert DNA spacers from λ bacteriophage. B. Schema of *In vitro* and *in vivo* analyses: MEL cells were transduced with various vectors to derive single copy MEL clones and hβ-globin expression and ChIP analysis was performed in differentiated clones. *In vivo* analysis was done using vector transduced Hbb^th3/+^ donor LSK cells transplanted into lethally irradiated Hbb^th3/+^ recipients and analyzed at 6 months post transplant. Secondary transplants were performed for CFU-S analysis. C. Representative FACS plot showing hβ-globin-expressing cells (% hβ+) for uninsulated (sBG, green) and insulated (sBG-I, Pink) single copy MEL clone with coefficient of variation (CV) of expression shown by arrows.

Self-inactivating lentivirus vectors were designed to incorporate either the 5′ 250 bp “core” (sBG^C^), two tandem repeats of the core (sBG^2C^), 5′ 400 bp (sBG^400^), 5′ 800 bp (sBG^800^) or the full-length 1.2 Kb cHS4 insulator (sBG-I). All vectors carried the human (h) β-globin gene and promoter and the locus control region enhancer. The different insulator fragments were cloned in the forward orientation into the U3 region of 3′ LTR, so that upon reverse transcription, integrated provirus in target cells has the insulated 3′ LTR copied to the 5′LTR, and flanks the hβ-globin expression cassette at both ends. To assess whether elements outside the 5′ 250 bp core merely provided a spatial scaffold, vectors with inert DNA spacers downstream of the core, sBG^400S^ and sBG^800S^, were also tested. All vectors were compared to the uninsulated control, sBG (**Figure 1A**).

First, MEL cells were infected with each of the lentivirus vectors and single integrant MEL clones were identified (**Figure 1B**). All analysis was performed only on single-copy MEL clones that carried hβ-globin and verified to have intact insulator sequences by PCR, and subjected to qPCR for vector copy number; hβ-globin expression was analyzed by FACS: 1) the percentage of hβ-globin expressing cells (% hβ+ cells) was used to determine chromosomal position effects, and 2) the variation of expression of hβ-globin expression in cells within a clone, as determined by the coefficient of variation (CV), was used to determine the clonal variegation in expression (**Figure 1C**). ChIP analysis was performed on the histones over the insulator regions and hβ-globin gene promoter in the different proviruses to study epigenetic modifications. Chromatin position effects of these vectors were confirmed *in vivo*, in RBC of Hbb^th3/+^ thalassemia mice transplanted with vector-transduced HSCs 24 weeks after transplant. Secondary transplants were then performed and single-integrant CFU-S following transplants were analyzed for hβ-globin protein and mRNA. In mice, hematological analysis, and HPLC for hβ-globin protein were additionally performed to quantify expression.

### Regions of cHS4 necessary to protect from chromatin position effects

**Figure 2:**
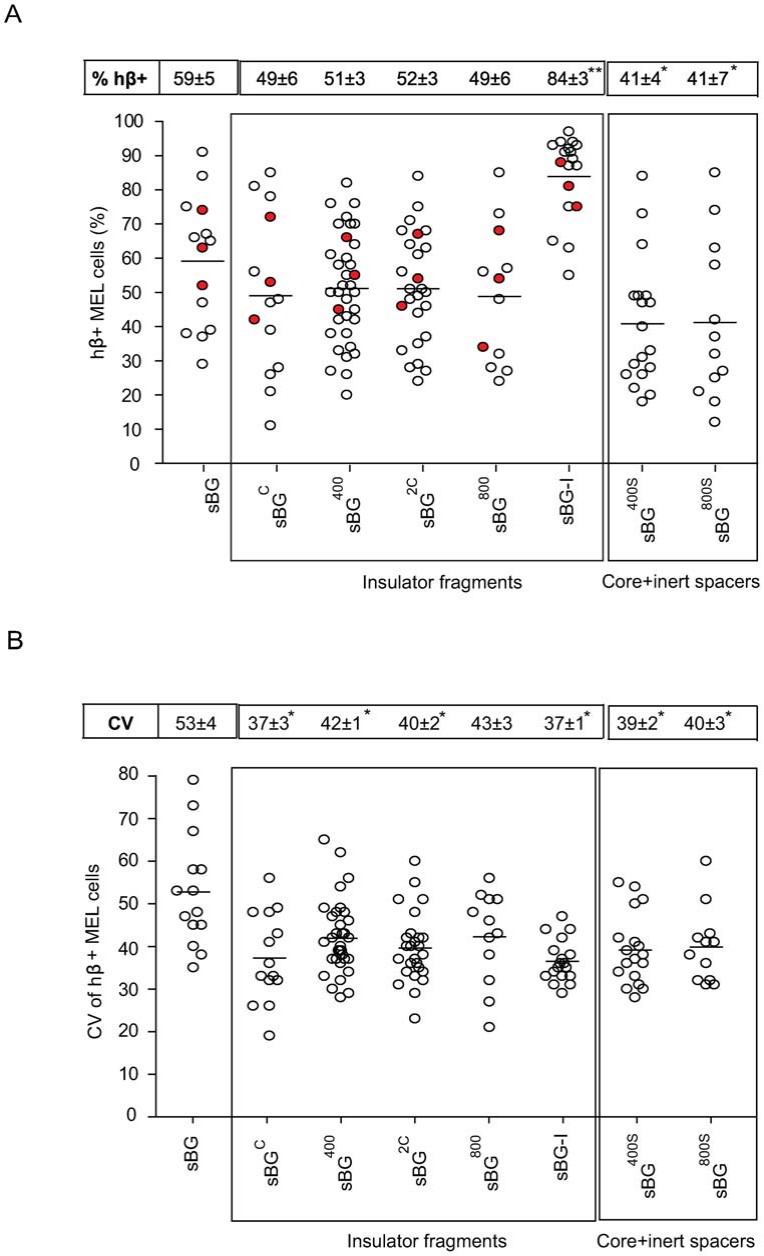
Human β-globin expressing cells in MEL clones. A. Proportion of hβ-globin-expressing cells (% hβ+) in MEL clones. Each circle represents an individual single copy MEL clone. B. CV values of hβ-globin expression of each clone. The means are represented with a horizontal line and the mean ± SEM of %hβ+ MEL cells and CV of hβ-globin expression for each vector are indicated in the box above. Filled circles represent representative clones picked for ChIP analysis. * P<0.05 by ANOVA, as compared to sBG.

Consistent with our previous results [21], a very high % of hβ+ cells were present in the sBG-I single-integrant clones compared to control sBG clones (P<0.01); the % of hβ+ cells in sBG^C^, sBG^2C^, sBG^400^ and sBG^800^ clones were not significantly different from the sBG control clones (**Figure 2A**). We ensured that presence of cHS4 in the LTR did not bias integration, and that our analysis was performed on distinct clones, by LM PCR and integration site sequencing on ten randomly selected sBG or sBG-I MEL clones. Insertions occurred near/in distinct genes between uninsulated and insulated clones, with no apparent bias (see Supplementary **Table S2**). Although there was no apparent integration bias between uninsulated and insulated single copy clones tested, the possibility that insulated vectors land in regions of the genome which are either more active or less prone to epigenetic silencing cannot be formally excluded. The presence of the cHS4 core (sBG^C^), or extended sequences of the insulator downstream to the core, up to 800 bp, did not increase the % hβ+ cells further; neither did tandem repeats of the core sequence, even though the latter has been shown to confer enhancer blocking effect in plasmid-based systems [25].

Another phenomenon seen with transgene expression is clonal variegation, defined as varying levels of expression in daughter cells with the same integration site. A quantitative way to determine clonal variegation is by FACS analysis of transduced clones and calculation of the coefficient of variation (CV) of expression of the transgene around the average expression of the transgene in the clone. The CV is a unit-less measure of variability calculated as ratio between sample standard deviation (SD) and the sample average. We observed a high CV in the uninsulated sBG clones (**Figure 2B**). The CV was significantly reduced in all vectors that contained the 5′ 250 bp core. These results were confirmed in clones derived from vectors that carried inert DNA spacers downstream of the core: sBG^400S^ and sBG^800S^, showing that the reduction in CV was specific to the insulator core, and in contrast to the data on % of hβ+ cells, which required the full-length insulator to be present.

It was notable that PCR for insulator sequences showed absence of the insulator sequences only in sBG^2C^ proviruses, with 6 of 24 clones (25%) MEL clones having both copies of the core deleted from both LTRs. We did not observe deletion of the insulator sequences in clones from all other vectors. Southern blot analysis of sBG^2C^ MEL pools confirmed deletion of one/both copies of the core in the majority of cells [24]. Reverse transcription of repeat sequences, known to result in recombination events in retroviral vectors [42], [43], [44], [45] likely caused unstable transmission of the vector with repeat core sequences.

**Figure 3:**
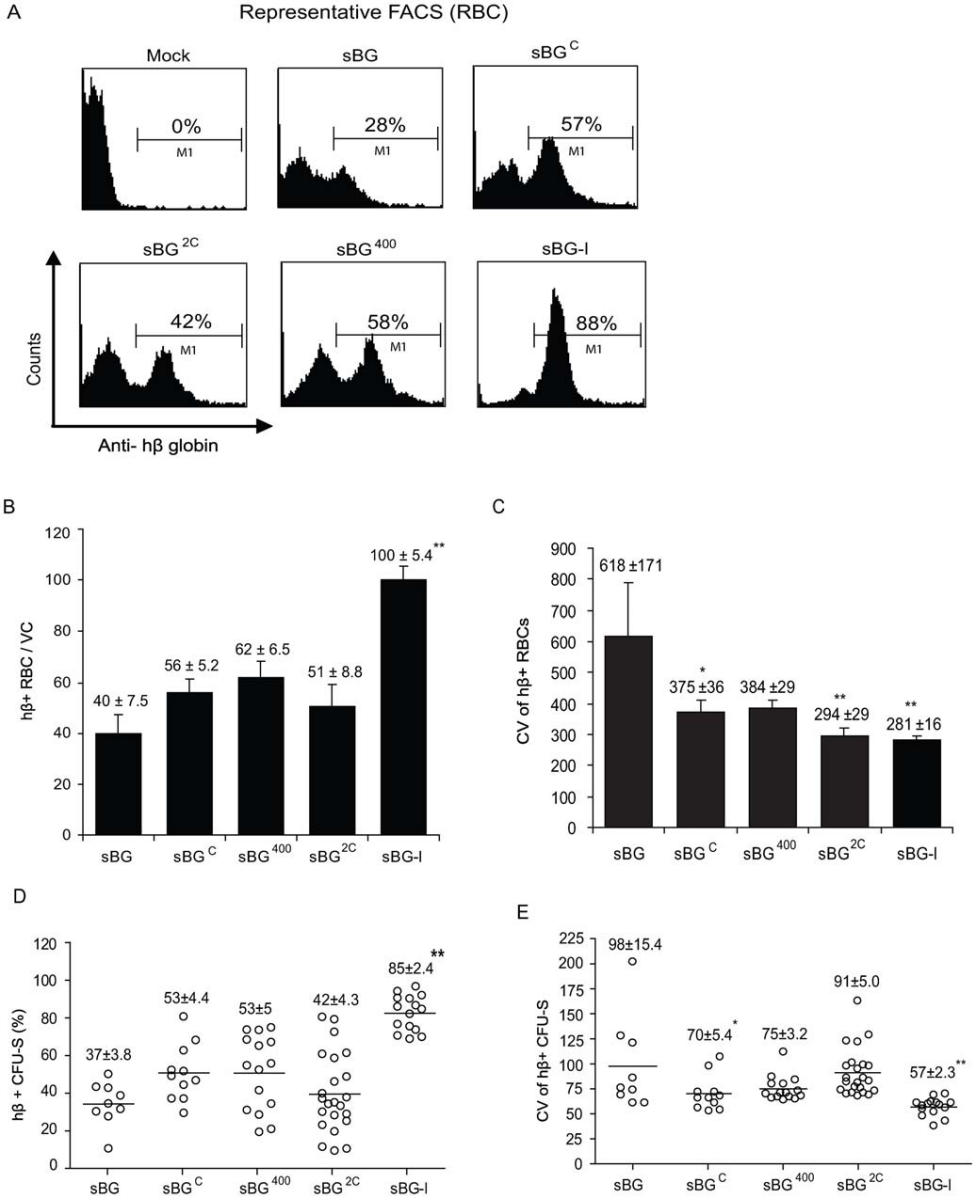
Human β-globin expression in RBCs and single copy secondary CFU-S. A. Representative FACS histograms showing (% hβ+ RBC are indicated within the histogram). B. Cumulative data on the percentage of hβ+ RBCs normalized to vector copy. C. The coefficient of variation (CV) of hβ expression in RBCs. D. Cumulative data on % hβ+ cells/CFU-S. Each circle represents an individual single integrant CFU-S. E. The CV of hβ expression in the individual CFU-S. Numbers above bar diagrams represent mean ± SEM and values significantly different from controls by ANOVA are marked by an asterisk. * P<0.05; ** P<0.01.

We confirmed this effect of the core versus the full-length cHS4 in vivo in thalassemia mice. Peripheral blood RBC were analyzed for hβ-globin expression 6 months following transplant. FACS analysis in RBC from sBG, sBG^C^, sBG^2C^, sBG^400^ and sBG-I groups of mice (representative plots shown in **Figure 3A**) shows that the % hβ+ RBC were significantly higher only in the sBG-I group of mice, compared to sBG group of mice, like the data in MEL cells; and the CV was significantly lower in all vectors that carried the core (P<0.01; **Figure 3B–C**). Taken together, our data suggest that the full-length cHS4 was required to shield against chromosomal position effects.

### Chromatin position effects in the clonal progeny of murine HSC following secondary transplants

The chromatin position effects were next confirmed in single copy secondary CFU-S. The secondary colony forming units-spleen (CFU-S) assay is considered the most stringent assay that is a ‘gold-standard’ for studying epigenetic effects of chromatin insulator elements in cells derived from hematopoietic stem cells. Notably, we did not observe any transduced CFU-S that was positive by PCR for vector-specific sequences that did not express hβ-globin by FACS, consistent with our results reported on lack of transgene silencing with erythroid-specific SIN lentivirus vectors [21], [46]. FACS analysis for (1) % hβ+ cells (representative plots in Supplementary **Figure S1**) and (2) TER-119 positive erythroblasts showed no difference in the percentage of TER-119+ cells between different vector groups (not shown). However, significantly higher % of hβ+ cells were only present in secondary CFU-S with the sBG-I vector. Again, the CV was significantly lower in CFU-S transduced with all the vectors carrying the core, compared to uninsulated sBG transduced CFU-S (**Figure 3D–E**). Real-time RT-PCR analysis on six randomly selected CFU-S from each group of mice showed that compared to the sBG vector, mRNA expression from the sBG-I CFU-S was approximately 2-fold higher (**Supplementary figure S2**). However, expression from sBG^C^, sBG^2C^ and sBG^400^ transduced CFU-S was not significantly different from that of sBG CFU-S. Taken together, these data suggest that the 5′ 250 bp core sequences in sBG^C^, sBG^400^, sBG^400S^, sBG^800^ and sBG^800S^ specifically reduced the clonal variegation of hβ-globin expression. However, the full-length cHS4 element was required for improved probability of expression from different integration events.

### Patterns of histone acetylation and methylation in the core region and the β-globin promoter region in insulated vectors

**Figure 4:**
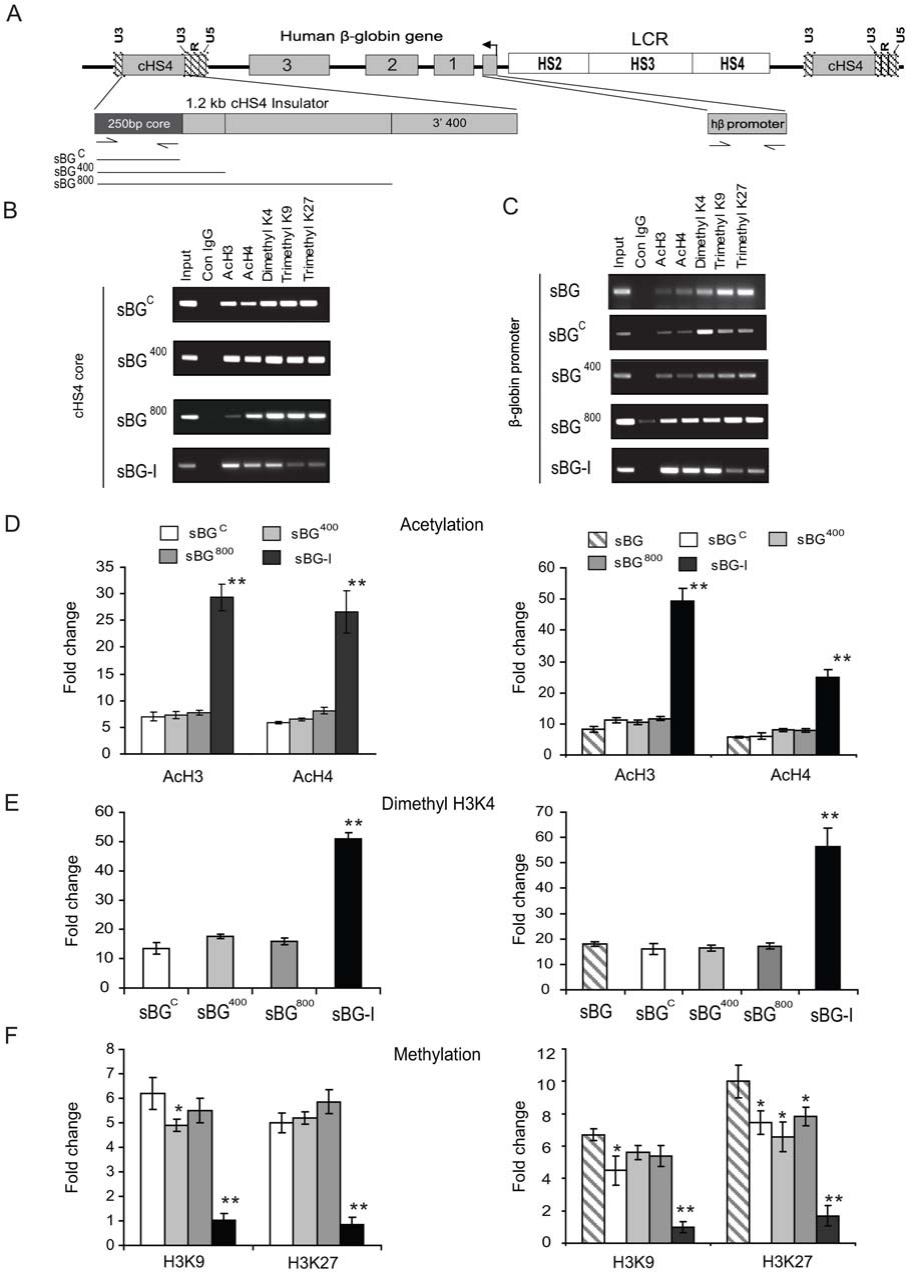
ChIP analysis showing the active and repressive histone marks on the 5′ 250 bp cHS4 core and the hβ promoter in MEL cell clones. A. Map of the proviral form of the vector. Arrows show the position of the primer pairs used for PCR and qPCR; and the lines represent insulator fragments. B–C. ChIP with antibodies against control IgG, acH3, acH4, H3K4-me2, H3K9-me3 and H3K27-me3 and semiquantitative PCR primers to the β-globin promoter region D–F ChIP with antibodies to AcH3 and AcH4 (D), H3K4-me2 (E); H3K9-me3 and H3K27-me3 (F) followed by qPCR using primers amplifying cHS4 core (left panels) and hβ-globin promoter (right panel) on pooled clones (shown in Figure 2A). *P<0.05; **P<0.01.

Next we determined the epigenetic modifications that accompany the specific effects seen with the various insulator regions by comparing the relative levels of active histone marks acH3, acH4 and H3K4me2 and repressive histone marksH3K9me3 and H3K27me3 between different proviruses in MEL clones. ChIP analysis was performed on the cHS4 core in three representative clones that were pooled together for each vector (clones chosen are shown as filled circles in **Figure 2A**) by semi-quantitative PCR (**Figure 4B–C**) and real-time PCR (**Figure 4D–F**). Clones carrying the sBG-I vector integrants showed approximately 6-fold enrichment of the active chromatin marks and decreased repressive chromatin marks over the cHS4 “core” fragment, compared to sBG^C^, sBG^400^ and sBG^800^, three vectors that carried the “core”.

We also analyzed for histone modifications over the hβ-globin promoter in the uninsulated vector (sBG) and all other vectors, which carried the “core”, to assess whether differences in histone patterns over the transgene promoter in vectors may have contributed to the reduced clonal variegation. There was a small but significant reduction in repressive chromatin patterns H3K27me3 with sBG^C^, sBG^400^ and sBG^800^ proviruses, compared to the uninsulated sBG provirus (**Figure 4F**, right panel). However, with the sBG-I provirus, where maximal insulator activity was present, the hβ-globin promoter region had markedly reduced repressive chromatin patterns.

These data show that the “core” sequences and extension of the core upto the 5′ 800 bp of cHS4 reduced activation marks over the transgene promoter to a small extent. However, a major reduction in repressed histone modifications over cHS4 and the transgene promoter region only occurred when the distal 3′ 400 bp sequences of cHS4 were present in addition.

### Hematological parameters in thalassemia mice transplanted with HSCs transduced with uninsulated and insulated vectors

**Figure 5:**
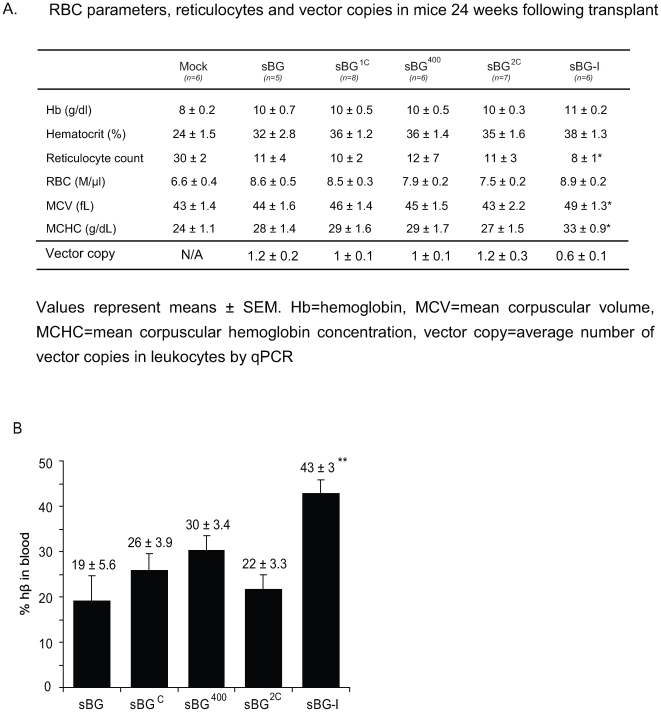
Human β-globin expression in mice. A. RBC parameters, reticulocytes and vector copies. Values represent means ± SEM. Hb = hemoglobin, MCV = mean corpuscular volume, MCHC = mean corpuscular hemoglobin concentration, vector copy = vector copies in leukocytes by qPCR. B. HPLC analysis of human β-globin protein in blood lysates as a percentage of total hemoglobin [hβ−mα / (hβ−mα + mβ−mα)]. Data is normalized to vector copy/cell in leukocytes. *P<0.05; **P<0.01.

The anemia, reticulocytosis and other RBC indices were improved even with the sBG vector (**Figure 5A**), consistent with published reports with uninsulated hβ-globin lentivirus vectors [17], [19], [47]. Hemoglobin of mock-transplanted mice was 7.7±0.2 gm/dL and the sBG group of mice was 10.4±0.7, with 1.2 vector copy per cell. It was noteworthy that the sBG-I group of mice had higher hemoglobin and the lowest reticulocyte count, despite having half the vector copies per cell compared to the sBG group of mice (hemoglobin 11±0.2 gm/dL; 0.6 vector copies per cell). When normalized for transduction efficiency, this amounts to a 5.2 gm increase in hemoglobin per vector copy in sBG-I mice over mock mice, in contrast to a 2.3 gm increase in hemoglobin per vector copy in the sBG mice. RBC parameters from the experimental mice showed significant improvement (**Figure 5A**; note that these data are not normalized for number of vector copies). Improvement in these indices was highest with the sBG-I mice, albeit not significantly, different unless normalized for vector copy.

HPLC analysis for hβ-globin protein in blood confirmed significantly higher hβ-globin expression only in the sBG-I mice: 43±3% of the total hemoglobin in RBC was derived from hβ-globin (hβ_2_mα_2_) in sBG-I mice as compared to 19±6% in the sBG mice, while that in sBG^C^, sBG^400^ and sBG^2C^ group of mice was not significantly different from control (**Figure 5B**). Human hβ-globin expression and hematological parameters in the sBG^2C^ group of mice were similar those seen in the uninsulated control group.

### Insulator activity in the 3′400 cHS4 region

**Figure 6:**
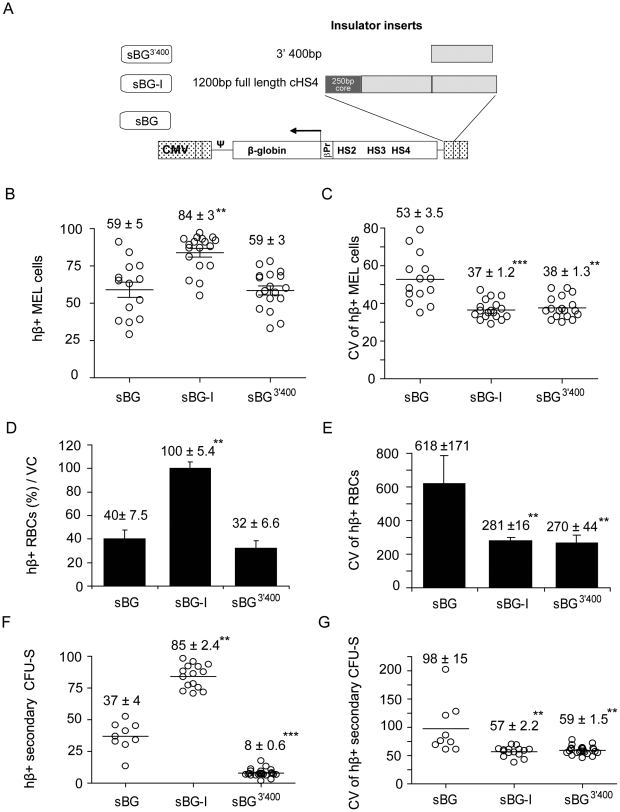
Effect of 3′400 bp region of the cHS4 insulator. A. Vector design of sBG^3′400^ vector. The full length cHS4 is shown for comparison. B–C. Proportion of hβ+ cells (B) and the coefficient of variation of hβ expression of sBG^3′400^ (C) in MEL clones. Each circle represents a single integrant MEL clone. The means are represented with a horizontal line and the mean ± SEM are represented in the figure. D–E. The percentage of hβ-globin+ RBC (D), and the CV of hβ expression (E) in mice. F–G. hβ-globin-expressing cells (F) and the CV of hβ expression (G) in single copy CFU-S following secondary transplant. Each circle represents individual CFU-S. Mean ± SEM and P-values are shown. * P<0.05; **P<0.01; *** P<0.001.

Since the 5′ 800 bp of cHS4 only reduced the CV, while full insulator activity was restored with the full-length 1.2 Kb insulator, we generated a vector carrying only the distal/3′ 400 bp region of the cHS4 (sBG^3′400^), derived MEL clones and transplanted mice with sBG^3′400^-transduced LSK cells. Note that unlike vectors described earlier, this vector does not contain the 5′250 bp “core” sequences (**Figure 6A**). The sBG^3′400^ vector had no effect on % of hβ+ cells in MEL clones or the % hβ+ RBC in mice (**Figure 6B,D**), an effect comparable to sBG clones, or those carrying the 5′ 250 bp “core” (sBG^C^). However, like all vectors carrying the 5′ core, sBG^3′400^ significantly reduced the CV of hβ-globin expression in MEL clones and in RBC (**Figure 6C,E**).

The amount of hβ-globin protein in the sBG^3′400^ mice, determined by HPLC analysis, was not significantly different from sBG (17.5±3% versus 19.5±5.6%), but was at least 2-fold lower than that seen in the sBG-I mice (43±3%; P<0.01) (**Figure 6F**). Overall, the 3′ 400 bp of cHS4 had activity that was very similar to the 5′ 250 bp core (**Figure 3**): it reduced clonal variegation, reflected in a reduced CV of hβ-globin expression in MEL clones and in RBC, but had no effect on the proportion of hβ-globin expressing red cells.

We confirmed the “core-like” effects of the 3′ 400 bp in individual single copy secondary CFU-S (**Figure 6G–H**), with results similar to those with the sBG^C^ vector (**Figure 3D–E**). The 3′ 400 region has no known consensus sequences for CTCF or USF-1, and this region has not been previously analyzed. It was noteworthy that neither the 5′ core, nor the 3′ 400 bp, when present alone, were able to improve the probability of expression of integrants/protect from position effects.

### Insulator activity of the 5′ “core” combined with the 3′ 400 bp

**Figure 7:**
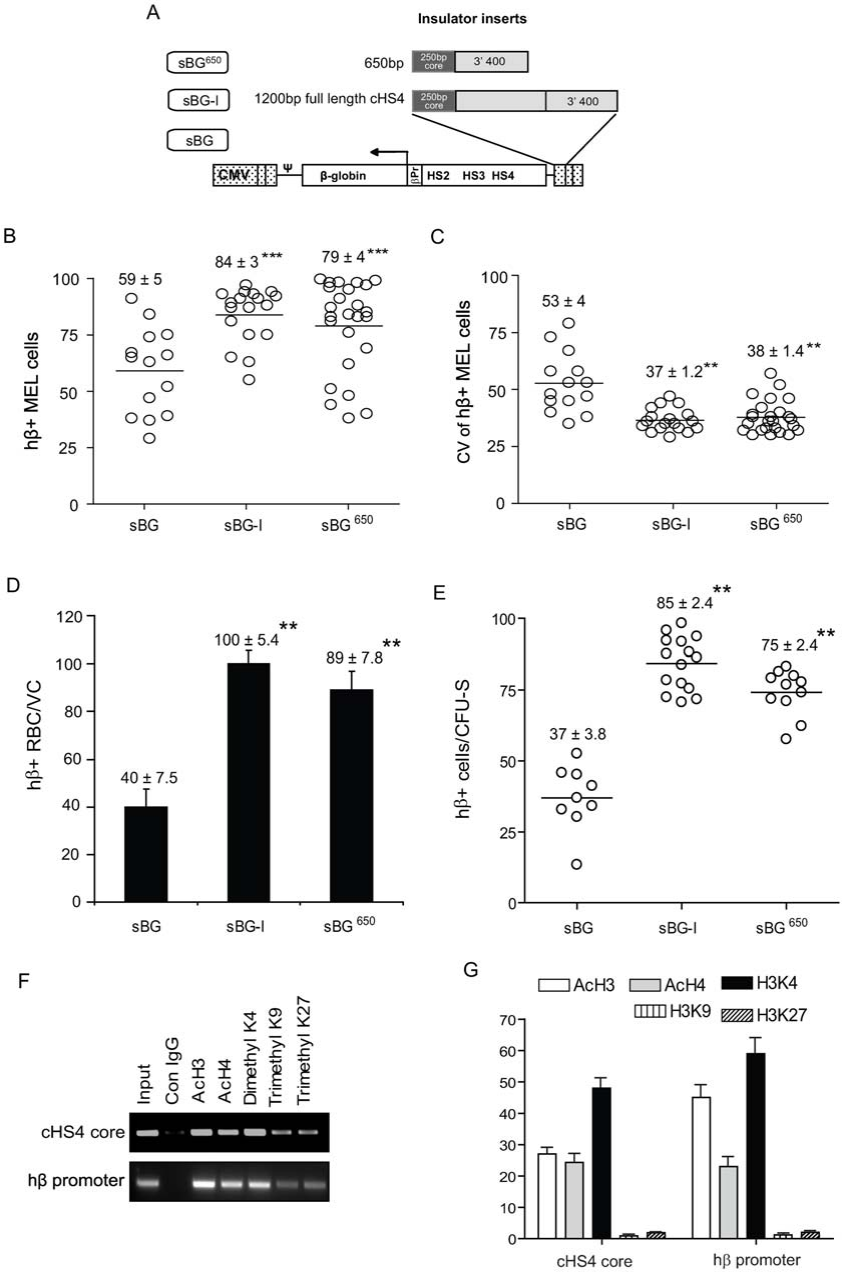
Effect of the combination of the 5′ core with the 3′ 400 bp regions of the cHS4 insulator. A. Vector design of sBG^650^ The full length cHS4 is shown for comparison. B. Proportion of hβ+ cells and C. CV of hβ-globin expression in sBG^650^ MEL clones. Each circle represents a single copy MEL clone. The means are represented with a horizontal line and the mean ± SEM is indicated above each group. D. Percentage of hβ-globin expressing RBC in ransplanted mice. E. Percentage hβ-globin expressing cells in single copy CFU-S from secondary mice. F–G. ChIP active and repressive chromatin followed by semiquantitative PCR (F) or qPCR (G) of the cHS4 core region or the hβ-globin promoter region.

When we combined the 5′ 250 bp core and the 3′ 400 bp sequences of cHS4 insulator (sBG^650^ vector; **Figure 7A**), this vector performed similar to the sBG-I vector - in MEL clones, in RBC of transplanted mice and in secondary CFU-S. The proportion of hβ-globin expressing cells in sBG^650^ MEL clones and RBC **(** **Figure 7B–D** **)** was significantly higher compared to sBG clones (P<0.001), and was similar to sBG-I clones. Likewise, the CV of the sBG^650^ clones was comparable to sBG-I clones (**Figure 7C**). The hβ-globin expression in the RBC of primary mice was comparable to sBG-I mice (**Figure 7D**). The amount of hβ-globin protein in the sBG^650^ mice, determined by HPLC analysis, was not significantly different from sBG-I mice (41±2.6% versus 43±3%, respectively), but was at least 2-fold higher than that seen in the sBG mice (19±6%; P<0.01). Five months after transplant, secondary transplants were performed to generate CFU-S, which confirmed that the sBG^650^ vector restored insulator activity similar to that seen with sBG-I vector (**Figure 7E**). The chromatin configuration over the core in sBG^650^ proviruses (**Figure 7F**) showed restoration of open chromatin patterns both over the insulator core and the β-globin promoter, identical to those seen in the sBG-I proviruses (**Figure 4**).

### Epigenetic modifications in the 3′400 bp region of cHS4 and its interaction with the core

**Figure 8:**
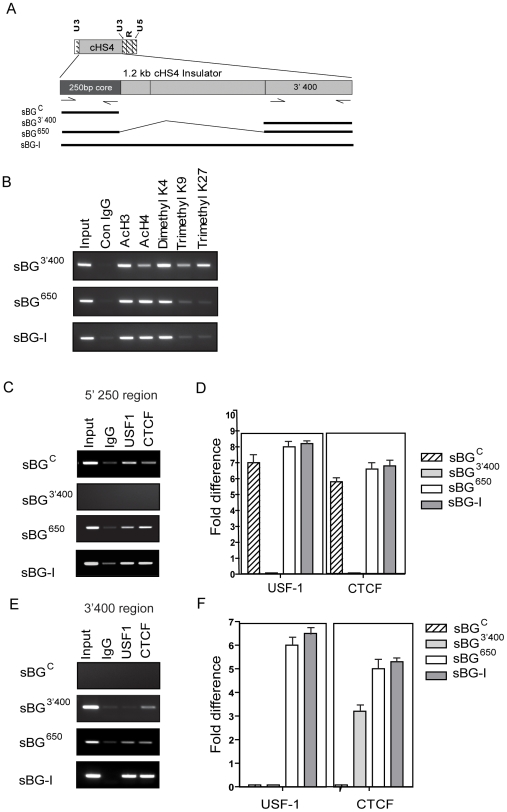
Chromatin patterns over the 3′400 bp and its interaction with the 5′ core region. A. A map of 3′LTR showing location of full length 1.2 kb insulator and the position of primers used in ChIP analysis . Vectors tested with the indicated regions of the cores are depicted beneath map B ChIP with antibodies to AcH3 and AcH4, H3K4-me2 and H3K9-me3 and H3K27-me3 followed by a semiquantitative PCR of the 3′400 region in sBG^3′400^, sBG^650^, sBG-I provirus. C–D ChIP with antibodies to USF-1 and CTCF followed by semi-quantitative PCR (C) or qPCR (D) for the core region. E–F ChIP with antibodies to USF-1 and CTCF followed by semi-quantitative PCR (C) or qPCR (D) for the 3/ 400 bp region of the sBG^C^, sBG^3′400^, sBG^650^ and sBG-I provirus in pools of three single copy MEL clones.

The chromatin configuration of the distal 3′ 400 bp portion of cHS4 have not been previously studied. We first analyzed the histone patterns over the 3′ 400 bp region (sBG^3′400^) when present alone (sBG^3′400^), or when in combination with the 5′ core (in sBG^650^ and sBG-I) (**Figure 8**). The acetylation and methylation patterns of the histones in the 3′400 region of sBG^3′400^ provirus (**Figure 8B**) were similar to those seen in the 250 bp core region in the sBG^C^ provirus (**Figure 4**). However, in sBG^650^ and sBG-I proviruses, the 3′ 400 bp sequences had increased acetylation marks and reduced repressive, showing once again, that the combination of the proximal and distal ends of cHS4 is necessary for open chromatin patterns. This effect was remininscent of the ChIP analysis over the 5′ core region or the β-globin promoter region in sBG-I (**Figure 4D and F**) or sBG^650^ (**Figure 7F and G**). Taken together, our genetic and epigenetic analysis suggested that the 5′ and 3′ ends of the insulator were functioning as two cores, which interacted for epigenetic modifications of chromatin on the insulator and promoter, to impart adequate insulator activity.

The 3′ 400 bp region, however, has no known CTCF or USF-1 motifs, that have been shown to impart enhancer blocking and barrier activity, respectively, to cHS4. It is conceivable; however that CTCF and/or USF-1 may perhaps be recruited to the 3′400 region. Using antibodies to USF-1 and CTCF, we immunoprecipitated chromatin from sBG^C^, sBG^3′400^, sBG^650^ and sBG-I proviruses from MEL clones. ChIP analysis was performed using semi-quantitative PCR and qPCR. When primers to the core region were used to amplify ChIP products, CTCF and USF-1 recruitment to the 5′ core region was evident (**Figure 8C–D**), as anticipated and shown previously [28], [48]. Interestingly, when 3′400 region primers were used to amplify the ChIP products, the sBG^3′400^ provirus showed enrichment for CTCF, albeit at somewhat lower levels than that seen over the core region. More notably, however, the sBG^650^ and sBG-I proviruses showed enrichment both USF-1 at the 3′ 400 bp region, an effect seen when both the proximal core and the distal 400 bp sequences were present. The 3′ 400 bp region, when present alone in sBG^3′400^, did not bind USF-1 (**Figure 8E–F**). These data suggest that the 3′ 400 bp region interacts with CTCF despite lack of the CCCTC consensus, which may explain the “core-like” activity in this region and the interaction between the 5′ core region and the 3′ 400 region of the cHS4 insulator (in sBG-I or sBG^650^) likely occurs via USF-1.

### Vector titers with the 650 bp cHS4 insulator

The 1.2 Kb cHS4 remarkably lowers titers of SIN-lentivirus vectors, limiting large-scale virus production for human trials. We have recently shown that the mechanism of reduction in titers is specifically due to the length of the insert in the 3′LTR [24]. Compared to sBG, sBG^650^ had very reasonable titers that were only 2.5±0.9 fold lower than sBG, in contrast to 10.4±2 fold lower titers of sBG-I (n = 3). Therefore, this optimized insulator can be used for the design of safer gene therapy vectors which would provide uniform and therefore higher expression and be scalable to large-scale production.

## Discussion

The full-length cHS4 insulator has been previously shown by us [17] and by others [18], [22], [49] to protect viral vectors against chromosomal position effects. The profound deleterious effects on viral titers however, have precluded its utility. Attempts to use only the 5′ 250 bp of cHS4, characterized to be the core of the insulator, have failed in viral vectors despite significant activity of the core in plasmid based systems, and loss of insulator activity with mutations in these regions [5], [14]. Two or more copies of the cHS4 core have been shown to have nearly the same insulator activity as the full-length cHS4 in plasmid transfections [25] but our data shows that these are unstable in lentiviral vectors. Southern blot analysis confirms that unlike sBG and sBG-I which are stably transmitted, the majority of sBG^2C^ proviruses recombine [24], and cannot be used effectively due to the inherent instability of repeat sequences in retroviruses. The sBG-I vector was stably transmitted, despite the presence of chicken repetitive-like elements (CR1) downstream of the cHS4 insulator core [50], [51]. Felsenfeld and colleagues showed that two copies of the core can dimerize, thereby connecting, clustering and tethering DNA domains to subnuclear/nucleolar structures, and forming “active chromatin hubs” via nucleophosphmin [32], [52], that have organized chromatin loops or scaffolds for transcription [27], [53].

Emery and colleagues have shown that extending the 5′ core by 150bp provides good insulator activity [5]. Of note, we did observe a slightly higher hβ-globin expression in RBC by HPLC with the presence of the core (sBG^C^) or the extended 150bp sequences beyond the core (sBG^400^), although the increase was not statistically significant. In their study, an uninsulated and 400 bp cHS4 insulated self-inactivating lentivirus vector was compared in MEL clones, but no comparisons were made to a lentivirus vector with full-length cHS4, which in our hands, shows the highest hβ-globin expression. We confirmed using stringent assays in vivo by extending the core to 400 and 800 bp, with no significant improvement in the probability of expression from different integration sites.

Regions surrounding the cHS4 insulator and β-globin promoter have been shown to constitutively higher marks of active chromatin in the native location [54]. The cHS4 prevents the spread of heterochromatin to the β-globin domain, even when adjacent heterochromatin domains have high repressive histone marks, H3K9me3 and H3K27me3 [55], [56]. Clones carrying the sBG-I vector integrants showed an enrichment of the active chromatin marks and a striking decrease in repressive chromatin marks over the cHS4 core compared to sBG^C^, sBG^400^ and sBG^800^ vectors, where no significant differences in these epigenetic marks were observed. Recently, Emery and coworkers have shown a 10-fold increased GFP expression from a 1.2Kb cHS4 insulated gamma-retroviral vector, which correlated with hyper-acetylation near the core and the promoter region [57], similar to what we observed while using full-length cHS4 insulator in lentivirus vectors.

Mechanistically, the USF-1/2 element in the insulator has been shown to recruit histone modifying enzymes to the core, and interact with histone lysine methyl transferase SET7/9 and p300/CREB-binding protein-associated factor (PCAF), thus increasing active chromatin marks. However, we observed no such increase in acH3, acH4 and H3K4me2 over the core or the 3′ 400bp when they flanked the transgene in the sBG^C^, sBG^400^, sBG^800^ and sBG^3′400^ vectors. This effect required the vector carrying the full length cHS4 (sBG-I, **Figure 4** **and** **8**) or both the core and 3′400bp combined sBG^650^ vector (**Figure 7** **and** **8**).

ChIP analysis over the hβ-globin promoter showed that compared to an uninsulated vector, the core alone reduced repressive chromatin marks over the promoter to some extent (**Figure 4F**), which may account for the reduction in CV from vectors carrying the core. However, the core was dependent on the 3′ 400 bp region and conversely, the 3′ 400 bp region dependent on the core for the high degree of histone acetylation and absent to minimal repressive marks over both these regions.

Models proposed to explain the effect of the cHS4 on surrounding chromatin include protection against transgene silencing by exclusion of methyl-CpG-binding proteins; indeed, cHS4 has been shown to block silencing by retroviral vectors [1], [40], [58], [59], [60], [61]. We did not observe any extinction of β-globin expression over time even with the uninsulated vector in mice, or MEL clones maintained up to 6 months in culture (data not shown) This may be due to several USF-1 elements in the β-globin LCR hypersensitive sites, that have been shown to interact with the E-box elements located in HS2 and in the β-globin gene promoter [62], [63]. It is conceivable that this resistance to silencing conferred by the LCR may override any activity we may have seen with the cHS4 core. Our results contrast those by Panell et al that retroviruses including those derived from HIV-1, dominantly silence a linked locus control region (LCR) beta-globin reporter gene in transgenic mice [64]. We have carefully looked for methylation and reported a lack of CpG methylation and extinction in expression with erythroid-specific SIN-lentivirus vectors *in vivo*, in primary and secondary recipients [46]. Our data suggests that in erythroid vectors, which otherwise resist silencing via promoter methylation [46], the full-length cHS4 was able to modify the histone patterns over the transgene promoter, and over itself to reduce position effects.

Intriguingly, the *in silico* analysis of the 3′ 400 bp region revealed no CTCF or USF1 binding sites, but sites for multiple known transcription factors. Any of these transcription factors, or perhaps a novel protein may be the interacting partner with the CTCF and/or USF-1. CTCF directly regulates the balance between active and repressive chromatin marks via binding to the cohesin complex [30], [65], [66], [67], [68], [69]. Our data reveals that the 3′ 400 bp region can also interact with CTCF: we were able to co-immunoprecipitate the 3′400 bp and CTCF from the sBG^3′400^ provirus (**Figure 8C–F**). The interaction was weaker that the interaction of CTCF with the core, suggesting this may have occurred indirectly via another protein bound to the 3′400 bp. However, this is merely speculation and remains to be tested. Interestingly, the 3′400 bp co-immunoprecipated with USF-1 antibody only when the 5′ core sequences were additionally present, suggesting that USF-1 likely forms a bridge between the 5′ and 3′ end of cHS4 to reduce position effects. Whether elements within the 3′ 400 bp recruit histone acetylases that bind USF-1 or cohesin and/or nucleophosphmin complexes to affect position effects would be important to determine.

In conclusion, in this study, we performed a systematic genetic and epigenetic analysis of insulator activity of the cHS4 *in vitro* and *in vivo* and identified novel “core-like” activity in the 3′ 400 bp. The 3′ 400 bp of cHS4, which contains no consensus sites for USF or CTCF, nevertheless binds CTCF, while USF-1 appears to bind and bridge the 5′ core and the 3′ 400 bp of cHS4. Our studies confirm and extend the observations by Felsenfeld and colleagues and others who have extensively characterized the 5′ 250 bp insulator core and identified CTCF and USF1/2 elements that contribute to insulator activity. The specific elements in the 3′ 400 bp that promote interaction with the 5′ 250 bp would be important to determine and may be present in other insulators in and across the genome/s. In the meanwhile, new vector systems flanked by this optimized ‘650 bp’ cHS4 sequences, can provide excellent insulation of the transgene without significant loss in viral titers and have important safety and efficacy implications for gene therapy.

## Materials and Methods

### Lentivirus Vectors

All vectors were obtained by cloning the different insulator fragments into NheI/EcoRV sites in the U3 3′LTR region of the lentivirus plasmid, as described [24]. This plasmid carried the human (h) β-globin gene and its regulatory elements (BG) [20], [21]. All insulator fragments were amplified by PCR using the insulator plasmid pJCI3-1 (kindly provided by Dr. Gary Felsenfeld, NIH, MD) and verified by sequencing, as described [24]. Cloning of the hβ-globin vector with and without the 1.2 kb cHS4 insulator has been described previously [20]. The sBG^1C^ vector was cloned by inserting EcoRI/XbaI 250 bp core insulator PCR product into sBG into BamHI/EcoRI restriction sites of the pBS plasmid. A second copy of the 250 bp core was then added into the pBS 1-core plasmid into EcoRI/KpnI sites, thus obtaining the pBS 2-core plasmid. The two tandem copies of the 250 bp core were then isolated digesting the pBS-2core plasmid with KpnI/XbaI, and then cloned into the sBG vector, obtaining sBG^2C^. The sBG^400^ and sBG^800^ vectors were obtained by cloning the 2 PCR products into the sBG NheI/EcoRV sites. The vectors containing DNA spacers were obtained amplifying different sizes of λ-phage DNA using the following primer combinations: spacerF1 and spacerR1, spacerF1 and spacerR2, amplifying 150 bp, 550 bp λ-DNA, respectively. ClaI/EcoRI digested PCR fragments were ligated into EcoRI/ClaI sites in the pBS-1 core plasmid, and 400 bp and 800 bp fragments from the pBS-1 core plasmid were restricted with HincII/XbaI and XbaI/XhoI, respectively, and cloned into NheI/EcoRV sites of sBG. Virus was produced by transient co-transfection of 293T cells [36] and titrated on MEL cells [20], [36].

### Cell Lines

MEL cells and 293T cells were maintained in DMEM (Mediatech, Inc) supplemented with 10% heat-inactivated fetal bovine serum (FBS; U.S. Bio-technologies, Inc.) and differentiated as described [17]. MEL cells were transduced to achieve less than 5% transduction efficiency for each of the vectors tested and cloned. Approximately 400 clones, derived from three independent transductions from each vector were screened by PCR for hβ-globin gene; positive clones were screened for an intact insulator region. Clones thus identified were then subjected to qPCR for single integrants [21], expanded and cryopreserved. An entire set of clones was thawed, differentiated and analyzed concurrently by FACS.

### Murine hematopoietic stem cell transduction and transplants

Hbb^th3/+^ thalassemia mice, a kind gift of Dr. Kaarin Gaensler (University of San Francisco, CA), were used for transplants. All animal studies were done using protocols approved by the Institutional Animal Use and Care Committee. Enrichment of lineage−Sca-1+c-*kit+* (LSK) hematopoietic stem/progenitor cells was performed on single cell suspension of bone marrow by immunomagnetic separation and FACS sorting (details in supplementary Materials and Methods S1) LSK cells were transduced in Stem Span (Stem Cell Technologies Inc, Vancouver, BC) with concentrated vector supernatants at an MOI of 10, twice at 12 h intervals as previously described [21]. 10,000 transduced LSK cells were co-transplanted with 2×10^5^ LK cells into 10.75Gy irradiated thalassemia recipients. CFU-S assay: Discrete spleen colony forming units (CFU-S) were dissected at day 12 after transplant of bone marrow cells from primary mice 24wk after transplant, as described earlier [17].

### Analysis for hβ-globin expression

Complete blood counts were performed on a Hemavet (Drew Scientific, Inc, Oxford, CT, USA). Reticulocyte count was analyzed by staining 1µl of whole blood with 200µl of Retic-COUNT reagent (BD Biosciences, CA) and enumerated on the FACSCalibur (BD). Quantitative analysis of hβ-globin protein in RBC was performed on hemolysates of blood by high performance liquid chromatography (HPLC), as previously described [37] and mRNA analysis quantified by real-time RT-PCR using validated primers and probes specific to hβ-globin (ABI Biosystems) using murine α-globin for normalization. FACS analysis following intracellular staining for hβ-globin was done as described before [17], [38].

### Chromatin immunoprecipitation (ChIP)

ChIP analysis was performed on MEL clones as described [39] with minor modifications (details provided in Supplementary material). Briefly, DNA samples from input and antibody-bound chromatin fraction were analyzed by qPCR using SYBR green (Applied Biosystems) using primer sets (Supplementary **Table S1**) in triplicate, and data analyzed as previously described [40]. The enrichment ratio was determined by calculating the ratio of DNA-ChIP to DNA-input and histone modification data normalized to the “no antibody” (IgG) control and primers corresponding to the necdin 5′ region and promoter region, as controls for repressed chromatin, to normalize the efficiency of immunoprecipitation. All the DNA-ChIP to DNA-input ratios were calculated as: 2^[Ct (Input) − Ct (ChIP)]^ divided with [dilution rate (ChIP)/dilution rate (Input)]. Ct values of all PCR products were determined by the SDS 1.2 software (Applied Biosystems). Mean and SEM values were determined for the fold difference, and two-tailed paired *t* tests to determine statistical significance (*p*<0.05).

### Integration site analysis

Ligation-mediated (LM) polymerase chain reaction was performed as described by Modlich et al [41] to map integration sites using primers and conditions described (Arumugam, Mol Ther 2009, in press citation).

### Statistical Analysis

Vectors were compared to the sBG vector Student's ‘t” test (unpaired and two tailed). ANOVA (Dunnett multiple comparison test) was also performed between groups for multiple comparisons. Data was expressed as mean ± SEM. P<0.05 was considered significant.

## Supporting Information

Figure S1 Representative histograms (FACS) showing hβ expressing cells in mock, sBG, sBGC, sBG2C, sBG400 and sBG-I single copy CFU-S. The % of hβ+ cells are indicated within the histogram.
(0.99 MB EPS)

Figure S2 Human β-globin messenger RNA (mRNA) expression in single copy secondary CFU-S of sBG, sBGC, sBG2C, sBG400 and sBG-I by qPCR. Murine α-globin expression served as the internal control against which hβ-globin expression was normalized. P values are shown in the figure. ** indicates P<0.01.
(0.87 MB EPS)

Table S1 The primers and probes used in chromatin immunoprecipitation (ChIP) is shown. ‘F’ represents forward primer and ‘R’ represents reverse primer.
(1.40 MB EPS)

Table S2 Insertional site analysis on single copy MEL clones from uninsulated sBG and insulated sBG-I vector with gene hits according to http://genome.ucsc.edu..
(0.37 MB PDF)

Materials and Methods S1 (0.03 MB DOC)
