# Supplementary material for: The 3′ Region of the Chicken Hypersensitive Site-4 Insulator Has Properties Similar to Its Core and Is Required for Full Insulator Activity
Source: PLoS One. 2009 Sep 10;4(9):e6995. doi: 10.1371/journal.pone.0006995 (PMC2736623; doi:10.1371/journal.pone.0006995)
Supplement: Table S2 — Insertional site analysis on single copy MEL clones from uninsulated sBG and insulated sBG-I vector with gene hits according to http://genome.ucsc.edu.. (0.37 MB PDF) [file pone.0006995.s004.pdf]

Table S2. Insertional Site Analysis on representative MEL clones

Clone #	Gene Symbol	Gene identification	Chromosome; Nucleotide position	Description
Insulated sBG-I vector				
1	Triap	BC006929	9; 107811858	TRAF interacting protein
2	Repeat element		19	
3	Prmt3	NM_133740	7; 49720743	Protein-Arginine methyl transferase
4	Rap1b	NM_024457	10; 117229934	member of RAS-oncogene family
5	2010310D06Rik	NM_172541	5; 136023764	Riken cDNA 2010310D06 gene
6	Repeat element		4	
7	Pdpn	BC026551	4; 142441749	Podoplanin encodes Type-1 integral membrane protein
8	Baz2b	NM_001001182	2; 59754076	Bromodomain adjacent to zinc-finger domain 2B
9	Repeat element		11	
10	Tmem57	NM_025382	4; 134092294	Transmembrane protein 57
Uninsulated sBG vector				
1	Akap 13	AK037815	7; 82854162	Ankyrin containing protein
2	Tcp1	NM_013686	17; 13117451	T- complex protein functions as a molecular chaperone
3	Cdkn3	AK010426	14; 46011478	Cyclin-dependent kinase associated protein
4	Plcb3	NM_08874	19; 7031156	Phospholipase C beta 3
5	Repeat element		3	
6	Dusp11	NM_028099	6; 85899687	Dual specific phosphatase functions in nuclear mRNA metabolism
7	ramp	NM_029766	1; 193373694	retinoic acid regulated nuclear matrix associated protein
8	Repeat element		1	
9	Rims1	NM_053270	1; 22730719	regulator of synaptic membrane exocytosis 1
10	Adam12	NM_007400	7; 141099540	a disintegrin and metalloprotease domain 12
