# Supplementary material for: The 3′ Region of the Chicken Hypersensitive Site-4 Insulator Has Properties Similar to Its Core and Is Required for Full Insulator Activity
Source: PLoS One. 2009 Sep 10;4(9):e6995. doi: 10.1371/journal.pone.0006995 (PMC2736623; doi:10.1371/journal.pone.0006995)
Supplement: Materials and Methods S1 — (0.03 MB DOC) [file pone.0006995.s005.doc]

**Supporting Materials and Methods**

ChIP analysis

Briefly, murine erythroleukemia clones were cross-linked with formaldehyde (1% final concentration) on ice for 10 min. The formaldehyde was neutralized with 2.5M glycine (final concentration 0.25M) for 10 min at 30ºC and centrifuged for 5 min at 2000g. Cells were washed sequentially with 1ml of ice-cold PBS, buffer I (0.25% Triton X-100, 10mM EDTA, 0.5mM EGTA, 10mM HEPES, pH 6.5), and buffer II (200 mM NaCl, 1 mM EDTA, 0.5 mM EGTA, 10 mM HEPES, pH 6.5). Cells were then resuspended in 0.3ml of lysis buffer (1% SDS, 10mM EDTA, 50mM Tris-HCl, pH 8.1, 1X protease inhibitor cocktail (Roche Molecular Biochemicals, Indianapolis, IN) and sonicated 10 times (20 sec ‘on’ followed by 40 sec ‘off’) at the maximum setting (Biorupter, Diagenode, NY) followed by centrifugation for 10 min on ice to produce 200-500bp fragments. Supernatants were collected and diluted 10 times in the ChIP dilution buffer (1% Triton X-100, 2 mM EDTA, 150 mM NaCl, 20 mM Tris-HCl, pH 8.1) followed by immuno-clearing with 40 μl of pre-blocked protein A-sepharose (Santa Cruz Biotech) with 2μg sheared salmon sperm DNA and pre-immune serum (1μg of rabbit serum with 10μl of 100mg/mL BSA for 2 hr at 4°C. A sample was retained for the preparation of the input sample.

Immuno-precipitation was performed for 6hr or overnight at 4°C with specific antibodies obtained from Upstate Biotechnology, Inc. (Lake Placid, NY):. Ab to diacetylated histone H3 (Catalog # 06-599), tetra acetylated histone H4 (Catalog # 06-866), dimethyl-histone K4, tri methyl-histone H3 K9, trimethyl-histone H3K27 and normal IgG. After immuno-precipitation, 40 μl protein A-Sepharose (pre-blocked with salmon sperm DNA) were added and further incubated for another 1 hr. Precipitates were washed sequentially for 10 min each in TSE I (0.1% SDS, 1% Triton X-100, 2 mM EDTA, 20 mM Tris-HCl, pH 8.1, 150 mM NaCl), TSE II (0.1% SDS, 1% Triton X-100, 2 mM EDTA, 20 mM Tris-HCl, pH 8.1, 500 mM NaCl), and buffer III (0.25 M LiCl, 1% NP-40, 1% deoxycholate, 1 mM EDTA, 10 mM Tris-HCl, pH 8.1). Precipitates were then washed three times with TE buffer and extracted twice with 1% SDS, 0.1 M NaHCO_3_. Eluates were pooled and heated at 65°C for a minimum of 6 hr to overnight to reverse the formaldehyde cross-linking,. DNA fragments were purified with a QIAquick Spin Kit (Qiagen, CA). Immuno-precipitation reactions were performed in triplicate using IgG as a non-specific control. Quantitative analysis of the active and repressive histone marks in the ChIP products from clones were assessed by quantitative real-time PCR. In order to normalize the efficiency of immuno-precipitation (IP), the normalization of chromatin IP was done using specific primers for necdin promoter region and 5’ region (which corresponds to a repressive chromatin region).

**Murine hematopoietic stem cell transduction and transplants** Hbb^th3/+^ thalassemia mice, a kind gift of Dr. Kaarin Gaensler (UCSF, CA), were used as donors and recipients. All animal studies were done using protocols approved by the Institutional Animal Use and Care Committee of Cincinnati Children’s Hospital Medical Center, Cincinnati, Ohio. Enrichment of lineage−Sca-1+c-*kit+* (LSK) hematopoietic stem/progenitor cells was performed on single cell suspension resuspended in IMDM containing 5% FBS. Low-density cells were enriched by equilibrium centrifugation over Ficoll-Histopaque (Sigma; density 1.077g/mL), washed, resuspended in IMDM/FBS and labeled with biotin-conjugated lineage antibodies CD5, CD8a, B220, Gr-1, Mac-1 and TER-119 (BD Pharmingen, San Diego, CA) according to manufacturer’s instructions. After 20 minutes of incubation at 4ºC, cells were washed and Dynabeads M-450 (Invitrogen) added a final ratio of 4 beads per cell. Cell/bead suspension was washed and exposed to magnetic depletion thrice in IMDM/FBS. The bead-free cells were then pre-incubated with FcγRII block (clone 2.4G2; BD Pharmingen) for 20 minutes at 4ºC and stained with anti-Ly6A/E-PE (Sca-1, rat IgG2a), anti-*c-kit*-APC (CD117) and streptavidin-FITC. Viable cells [7-AAD(-)] were sorted as LSK cells on FACS Vantage. The 7-AAD(-) lineage^-low^*c-kit^+^* (LK) cells were also collected.

# LSK cells were transduced in 1mL Stem Span (Stem Cell Technologies Inc, Vancouver, BC) with concentrated sBG, sBG-I, sBG^C^, sBG^2C^ and sBG^400^ supernatants at an MOI of 10, twice at 12h intervals, in medium containing recombinant cytokines as previously described [21]. 10,000 transduced LSK cells were co-transplanted with 2x10^5^ LK cells into 10.75Gy irradiated thalassemia recipients.
